# Clinicopathological parameters for circulating tumor DNA shedding in surgically resected non-small cell lung cancer with EGFR or KRAS mutation

**DOI:** 10.1371/journal.pone.0230622

**Published:** 2020-03-20

**Authors:** Min-Sun Cho, Chul Hwan Park, Sungsoo Lee, Heae Surng Park

**Affiliations:** 1 Department of Pathology, Ewha Womans University Seoul Hospital, Ewha Womans University College of Medicine, Seoul, Korea; 2 Department of Radiology, Research Institute of Radiological Science, Gangnam Severance Hospital, Yonsei University College of Medicine, Seoul, Korea; 3 Department of Thoracic and Cardiovascular Surgery, Gangnam Severance Hospital, Yonsei University College of Medicine, Seoul, Korea; Baylor College of Medicine, UNITED STATES

## Abstract

**Background:**

Circulating tumor DNA (ctDNA) is cell-free DNA that is released into peripheral blood by tumor cells. ctDNA harbors somatic mutations and mutant ctDNA obtained from blood can be used as a biomarker in advanced non-small cell lung cancer (NSCLC). In this study, we investigated the clinicopathological properties of tumors that shed ctDNA in surgically resected NSCLC patients.

**Methods:**

Consecutive cases of NSCLC with matching surgically resected tissue specimens and peripheral or specimen blood samples were eligible for this study. EGFR and KRAS mutations in plasma ctDNA and formalin-fixed paraffin-embedded tissue were analyzed using peptide nucleic acid clamping-assisted method. The plasma and tissue results were compared according to clinicopathological features.

**Results:**

Mutation analyses were available for 36 cases. EGFR and KRAS mutations were present in 41.7% (15/36) and 16.7% (6/36) of tissue samples, respectively. Among EGFR and KRAS-mutant tumors, plasma mutation detection sensitivity was 13.3% (2/15) for EGFR and 33.3% (2/6) for KRAS. The presence of ctDNA in plasma was significantly associated with higher pathological tumor stage (*p* = 0.028), nodal metastasis (*p* = 0.016), solid adenocarcinoma pattern (*p* = 0.003), tumor necrosis (*p* = 0.012), larger primary tumor diameter (*p* = 0.002) or volume (*p* = 0.002), and frequent mitosis (*p* = 0.018) in tissue specimens. All tumors larger than 4 cm in maximal diameter or 25 cm^3^ in volume shed ctDNA in plasma. In subgroup analysis among EGFR mutated adenocarcinoma, ctDNA was significantly associated with nodal metastasis (*p* = 0.029), vascular invasion (*p* = 0.029), solid adenocarcinoma pattern (*p* = 0.010), and tumor necrosis (*p* = 0.010), high mitotic rate (*p* = 0.009), large pathological tumor size (*p* = 0.027), and large tumor volume on CT (*p* = 0.027).

**Conclusion:**

We suggest that primary or total tumor burden, solid adenocarcinoma morphology, tumor necrosis, and frequent mitosis could predict ctDNA shedding in pulmonary adenocarcinoma.

## Introduction

Identification of oncogenic drivers and development of targeted therapies has changed treatment algorithms for advanced non-small cell lung cancer (NSCLC). Molecular testing for EGFR, ALK, and ROS1 is currently mandatory for patients with lung cancer in routine practice [1]. So far, tissue genotyping is considered the gold standard for detecting genetic alterations in tumors. Unfortunately, tumor tissue is not adequate for molecular testing in 20–30% of NSCLC patients at diagnosis [2, 3]. Moreover, rebiopsy is not always feasible or biopsy tissue is insufficient to allow molecular testing in NSCLC patients with disease progression on treatment [4]. Plasma contains tumor-derived, extracellular DNA (circulating tumor DNA, [ctDNA]) and plasma genotyping could be a suitable substitute for mutation analysis when tumor tissue is unavailable. However, the fraction of ctDNA in the blood is very low and the sensitivity of plasma genotyping remains a challenge despite continued development of highly sensitive ctDNA assays [5].

The sensitivity of plasma tests depends on not only preanalytical and analytical factors but also the rate of ctDNA release from the tumor, so-called “ctDNA shed”. Tumors not shedding ctDNA most likely have false negative result in plasma, even the tumor have targetable mutation. ctDNA is thought to be released into the plasma when tumor cells are going through necrosis or apoptosis [6]. ctDNA shedding is related to tumor size, necrosis, and the vascularity of tumor [6, 7]. However, comprehensive histopathological features of shedding tumors in NSCLC was not evaluated. Most liquid biopsy studies have been performed in advanced NSCLC. In advanced stages, only a small biopsy specimen is taken, so sampling bias may arise when evaluating histopathological features of shedding tumors. Herein, we examined the histopathology of entire sections of primary lung tumor to assess the histopathological features of ctDNA shedding tumor.

Several recent studies performed next generation sequencing (NGS) of plasma in surgically resected lung cancer [8, 9]. Abbosh *et al*. studied clinicopathological predictors of ctDNA including clonal/subclonal and driver/passenger mutations [9]. Chen *et al*. correlated clinicopathological factors with ctDNA level in plasma without discriminating mutation type [8]. EGFR and KRAS are the most commonly mutated oncogenes involved in the pathogenesis of NSCLC. EGFR mutations can predict response to tyrosine kinase inhibitors (TKIs) such as gefitinib and erlotinib [10], while KRAS mutations are associated with poor response to TKIs and worse prognosis [11]. Therefore, we focused on detection of EGFR and KRAS mutations in plasma, which is more clinically relevant.

The objectives of this study were: 1) to compare EGFR and KRAS mutation status between plasma and tumor tissue in surgically resected TKI-naïve NSCLC; 2) to compare histopathological features between ctDNA shedding and non-shedding tumors; and 3) to evaluate parameters predicting ctDNA shedding.

## Materials and methods

### Case selection

**Fig 1:**
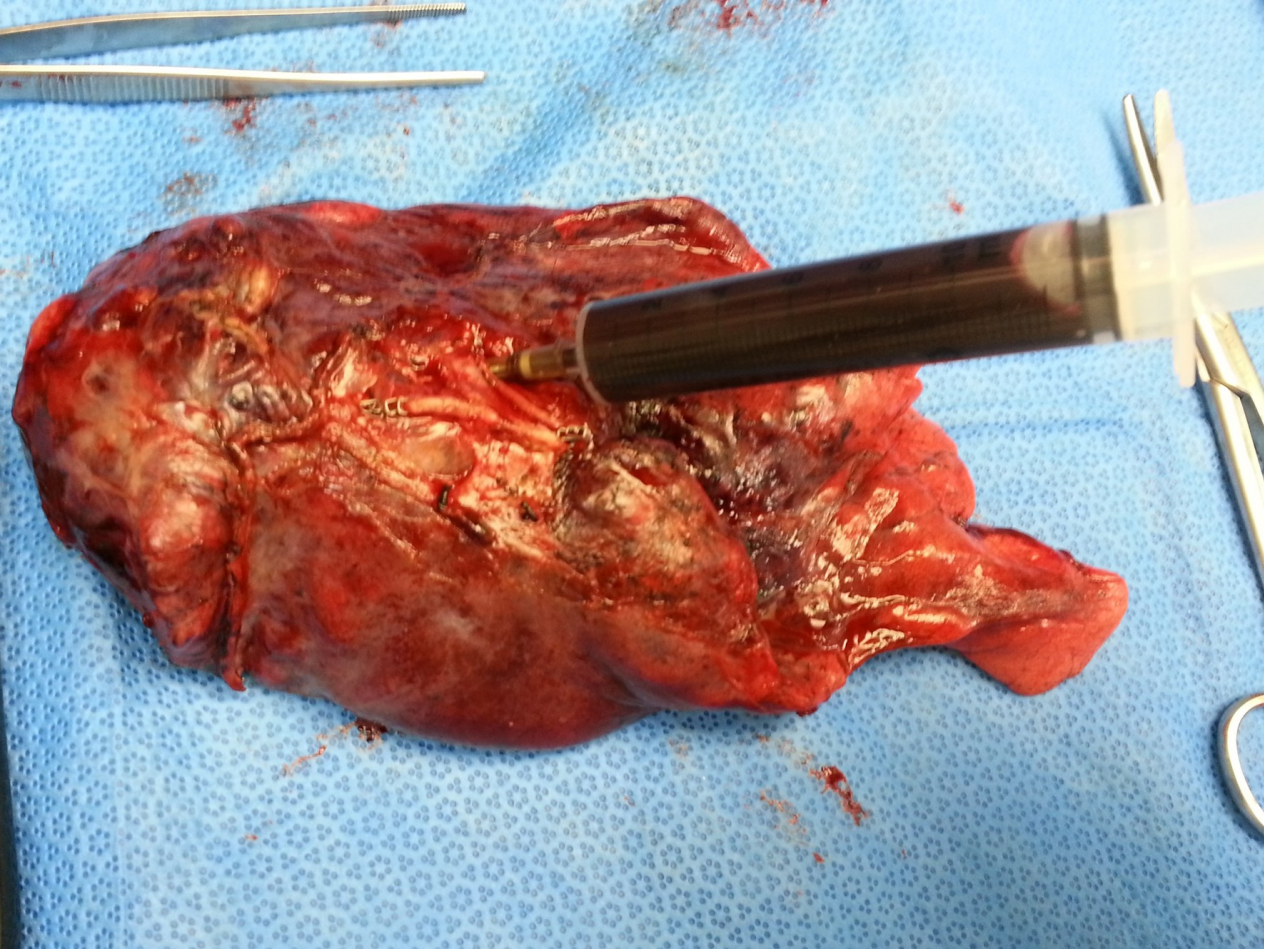
Blood sampling from lobectomy specimen. When a lobectomy specimen of fresh lung tissue was submitted to the Department of Pathology to evaluate bronchial resection margin during surgery, peripheral blood was obtained from a pulmonary vessel.

Consecutive cases of NSCLC in patients who underwent surgical resection at Gangnam Severance Hospital (Seoul, South Korea) between May 2016 and October 2017 and had a matching plasma sample in the biobank were eligible for enrollment in this study. All peripheral blood samples were obtained within 24 hours just prior to surgery and immediately processed to isolate plasma. In cases of lobectomy specimen, pulmonary vessel puncture and blood sampling was done during frozen sectioning (Fig 1) and plasma was isolated within several hours. Whole blood was collected in BD Vacutainer^TM^ K2 EDTA tubes (BD Bioscience, CA, USA) and centrifuged for 10 min at 1000 x g and 4°C temperature with break off setting. Without disturbing the buffy coat layer or red blood cells, plasma supernatant was aspirated and transferred to new tubes, then further centrifuged for 10 min for 2000 x g and 4°C with break off setting. The supernatant was transferred to new tubes without disturbing the pellet. The plasma was stored frozen at -80°C at the depository of Gene Bank, Yonsei University, Gangnam Severance Hospital, until use. Tumor tissue samples were collected during surgery. Matched tumor tissue and plasma samples were obtained from Gene Bank. This study protocol was approved by the Institutional Review Board of Gangnam Severance Hospital (protocol no: 3-2017-0008) and the requirements for informed consent was waived.

A total of 36 NSCLCs were finally selected. The clinicopathological characteristics including the age and sex of the patients, operation type, maximal tumor diameter, tumor volume on chest CT, histologic subtype according to 2015 World Health Organization (WHO) classification, lymphatic, vascular and perineural invasion, pT and pN classification, and TNM stage were investigated. pT and pN classification and TNM stage were determined based on the 8^th^ edition of the American Joint Committee on Cancer staging system.

### Histopathological evaluation

Cytological atypia was classified into three categories as follows: mild, relatively uniform nuclei with indistinct nucleoli at x100 magnification; moderate, relatively uniform nuclei with distinct nucleoli at x100 magnification; severe, bizarre, enlarged nuclei of variable sizes. For heterogeneous tumors, the highest degree of atypia was recorded.

For mitotic rate, we examined 10 high power fields (HPFs) using an Olympus BX41 microscope at x400 magnification (objective, x40; visible area, 2.37mm^2^) in the most mitotically active area.

### Measurement of tumor volume

Tumor volume was retrospectively measured on preoperative chest CT images. The mean time interval between CT scan and surgery was 29 days (range, 0–105). CT scans of the chest were performed using one of the following scanners: Somatom Sensation 16, Somatom Sensation 64, Somatom Definition AS+ (all Siemens Medical Solutions, Erlangen, Germany), or Brilliance 64 CT (Philips Healthcare, Best, The Netherlands). In the supine position, CT images were obtained from the lung apex to the adrenal glands at the end of inspiration. CT parameters were as follows: 120 kVp, 50–130 mAs, and 1–3-mm scan thickness at 1–3-mm intervals. A board-certified chest radiologist with 10 years of experience (CHP) measured tumor volume, blinded to the pathologic results. For volumetric analysis, all axial CT images were analyzed using a commercially available reconstruction program (Aquarius iNtuition^™^ Ver.4.4.6; TeraRecon, Foster City, CA, USA). Each tumor margin was manually drawn by a radiologist on every axial CT image in the lung window setting and three dimensional tumor area was determined by integrating all axial images. The tumor volume was calculated automatically by adding the volumes of all voxels within the segmented tumor area.

### Mutation analysis for tissue

Genomic DNA was extracted from 10% neutral-buffered formalin-fixed, paraffin-embedded tumor tissue using the Maxwell 16 FFPE Plus LEV DNA Purification kit (Promega, Manheim, Germany) according to manufacturer’s instruction. DNA was quantified using a fluorescence assay (Invitrogen) and 10–25 ng of DNA was used for mutation analysis. The PANAmutyper^TM^ R EGFR and KRAS detection kit (PANAGENE Inc., Daejeon, South Korea) was used for mutation detection. PANAmutyper^TM^ R is a PCR-based multiplex assay that uses peptide nucleic acids (PNA) that complementarily bind to the wild-type DNA and suppress its amplification. Therefore, a very small amount of mutant DNA can be selectively amplified and detected with high sensitivity: the limit of detection is between 0.01% and 0.1% when using 2 mL of plasma. PANAmutyper^TM^ R can be used both in plasma and tissue. PANAmutyper^TM^ R is an in vitro diagnostic test which qualitatively detect defined EGFR and KRAS mutations. The equality of detecting EGFR mutation between PANAmutyper^TM^ R EGFR and Roche cobas^®^ EGFR mutation test v2 was proved. Korean Ministry of Food and Drug Safety approved PANAmutyper^TM^ R EGFR as a companion diagnostic test to select patients for erlotinib or osimertinib. The PANAmutyper^TM^ R EGFR kit can detect EGFR mutations, such as G719X, exon 19 deletions, T790M, S768I, exon 20 insertion, L858R, L861Q, and C797S. PANAmutyper^TM^ R KRAS kit can detect point mutations in codon 12, 13, 59, 61, 117, and 146 of the KRAS gene.

### Mutation analysis for plasma

Circulating cell-free (cfDNA) was extracted from 2 mL of plasma using the QIAamp Circulating Nuclei Acid Kit (Qiagen, Hilden, Germany) according to the manufacturer’s protocol. The PANAmutyper^TM^ R EGFR and KRAS detection kit was used for mutation detection. Quantification of ctDNA extracted from plasma was omitted. DNA extracted from tissue exists in large amount, so suppression of wild type DNA by clamp probe is not sufficient and false positive result would be possible. However, ctDNA exists in a very small level and adequate volume of plasma containing ctDNA (required volume in PANAMutyper: 2mL) should be used. If the quality or quantity of ctDNA in plasma sample is not adequate, real time PCR cannot be carried out. In this study, all plasma samples were successfully amplified.

### Statistical analysis

The Fisher’s exact and Mann-Whitney *U* tests were used for categorical and continuous variables to examine the correlation between the presence of ctDNA and each clinicopathological parameter. A *p* value of <0.05 was considered statistically significant. All statistical analyses were performed using SPSS software version 12.0 (SPSS, Chicago, IL, USA) and MedCalc version 18.11.6. (MedCalc Software, Mariakerke, Belgium) for Windows.

## Results

### Clinicopathological characteristics

The baseline characteristics of the patients are summarized in Table 1. Most patients (35 out of 36) had a primary tumor and one patient had a recurrent tumor. Two patients, who had neither EGFR nor KRAS mutation in their primary lung tumors had undergone neoadjuvant chemotherapy before surgery. All patients were naïve to TKI treatment before surgical resection of the tumor. All cases were ALK gene rearrangement negative.

**Table 1:** Baseline characteristics of non-small cell carcinoma (n = 36).

Parameters	
Age (range)	66 (33–81)
Sex	
Male	25
Female	11
Tumor size (cm)	
Average	3.4
Range	0.8–10.5
Tumor volume measured from chest CT (cm^3^)	
Average	25.0
Range	0.5–201
Operation type	
Wedge resection	3
Lobectomy	33
Histologic classification	
Invasive nonmucinous adenocarcinoma	
Lepidic predominant	3
Acinar predominant	10
Papillary predominant	7
Solid predominan	5
Invasive mucinous adenocarcinoma	2
Squamous cell carcinoma	6
Large cell neuroendocrine carcinoma	1
Mucoepidermoid carcinoma	1
pT classification*	
pT1	17
pT2	9
pT3	5
pT4	4
pN classification*	
pN0	28
pN1	2
pN2	4
pNx	2
Pathological TNM stage*	
I	23
II	5
III	6
IV	1
Recurrent	1
EGFR mutation	
Mutant	15
Wild	21
KRAS mutation	
Mutant	6
Wild	30

*Stages adapted from the American Joint Committee on Cancer TNM staging system, 8th edition

### EGFR and KRAS mutations in tumor tissue and plasma

Detailed EGFR and KRAS mutation results for matched plasma and tissue samples are presented in Table 2. In 19 out 36 patients, both peripheral and specimen blood were available and showed the same results. In 17 patients, either peripheral or specimen blood were available. EGFR and KRAS mutations in tissue was detected only in adenocarcinomas. Concurrent mutations of T790M and L858R were present in one case, while C797S was not detected in any cases. Among adenocarcinomas, EGFR and KRAS mutations were present in 55.6% (15/27) and 22.2% (6/27) of tissue samples, respectively. Comparison of EGFR and KRAS mutation status between tissue and plasma was summarized in Tables 3 and 4. The concordance rate between tissue and plasma samples was 63.9% in EGFR and 88.9% in KRAS. Among EGFR or KRAS- mutant tumors, the mutation detection sensitivity of the plasma sample was 13.3% (2/15) for EGFR and 33.3% (2/6) for KRAS. The overall sensitivity of detecting driver mutation in plasma was 19.0%. The mutation detection specificity of plasma was 100% for both EGFR (21/21) and KRAS (30/30).

**Table 2:** Detailed results of EGFR and KRAS mutations in matched plasma and tissue samples.

Histologic subtype	Stage	Case No.	EGFR mutation			KRAS mutation		
			Peripheral blood	Specimen blood	Tissue	Peripheral blood	Specimen blood	Tissue
ADC	I	1	n/a	wt	wt	n/a	wt	wt
		5	wt	n/a	L858R	wt	n/a	wt
		6	wt	wt	wt	wt	wt	wt
		8	wt	wt	L858R T790M	wt	wt	wt
		9	wt	wt	wt	wt	wt	wt
		10	n/a	wt	wt	n/a	wt	wt
		11	n/a	wt	L858R	n/a	wt	wt
		15	wt	wt	E19del	wt	wt	wt
		18	wt	wt	wt	Wt	wt	G12V
		22	wt	wt	E19del	wt	wt	wt
		24	wt	n/a	wt	wt	n/a	G12A
		26	wt	wt	L858R	wt	wt	wt
		28	wt	n/a	L858R	wt	n/a	wt
		29	wt	n/a	E19del	wt	n/a	wt
		30	wt	n/a	E19del	wt	n/a	wt
		32	wt	n/a	E19del	wt	n/a	wt
		33	wt	n/a	L858R	wt	n/a	wt
		36	wt	wt	E19del	wt	wt	wt
	II	16	n/a	wt	wt	n/a	wt	wt
		31*	wt	wt	wt	wt	wt	wt
		35	L858R	n/a	L858R	wt	n/a	wt
	III	4	wt	wt	wt	G12D	G12D	G12D
		12	wt	wt	G719A	wt	wt	wt
		13	n/a	wt	wt	n/a	G12V	G12V
		14†	n/a	wt	wt	n/a	wt	G12D
		17	n/a	wt	wt	n/a	wt	wt
		19	L858R	L858R	L858R	wt	wt	wt
	IV	34	wt	n/a	wt	wt	n/a	Q61H
SqCC	I	7*	wt	wt	wt	wt	wt	wt
		21	wt	wt	wt	wt	wt	wt
		27	wt	wt	wt	wt	wt	wt
		20	wt	wt	wt	wt	wt	wt
	II	2	n/a	wt	wt	n/a	wt	wt
		25	wt	wt	wt	wt	wt	wt
MEC	I	3	wt	wt	wt	wt	wt	wt
LCNEC	III	23	wt	wt	wt	wt	wt	wt

ADC, adenocarcinoma; SqCC, squamous cell carcinoma; MEC, mucoepidermoid carcinoma; LCNEC, large cell neuroendocrine carcinoma; wt, wild type; n/a, not applicable

* cases with neoadjuvant chemotherapy

† recurred case

**Table 3:** Comparison of EGFR mutation status between matched tissue and plasma samples.

	Tissue EGFR mutation		Subtotal
	Mutant	Wild type	
ctDNA EGFR mutation			
Mutant	2	0	2
Wild type	13	21	34
Subtotal	15	21	36

ctDNA, circulating tumor DNA

**Table 4:** Comparison of KRAS mutation status between matched tissue and plasma samples.

	Tissue KRAS mutation		Subtotal
	Mutant	Wild type	
ctDNA KRAS mutation			
Mutant	2	0	2
Wild type	4	30	34
Subtotal	6	30	36

ctDNA, circulating tumor DNA

### EGFR and KRAS mutation detection in plasma and its clinicopathological correlation

**Fig 2:**
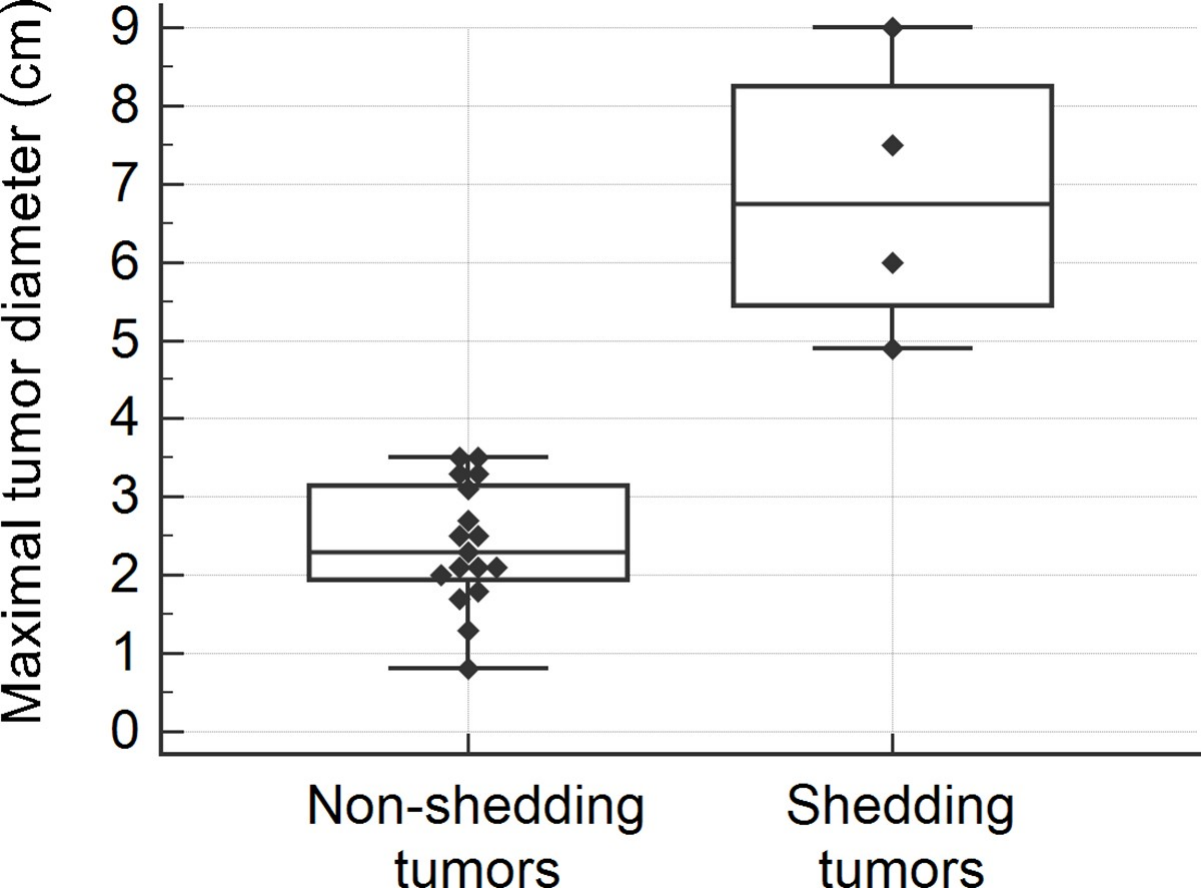
Box and whisker plot of pathological tumor size in non-shedding and shedding tumors.

**Fig 3:**
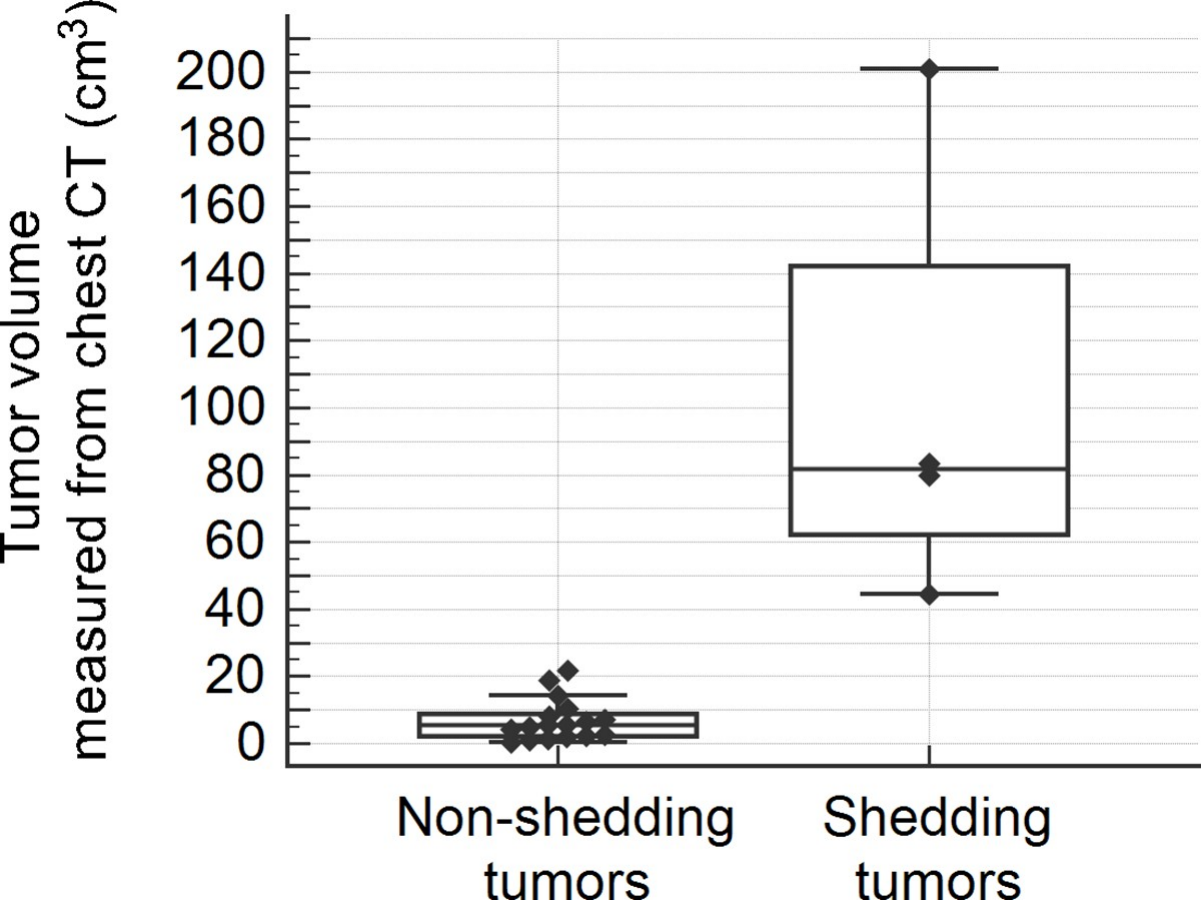
Box and whisker plot of tumor volume measured from chest CT in non-shedding and shedding tumors.

**Fig 4:**
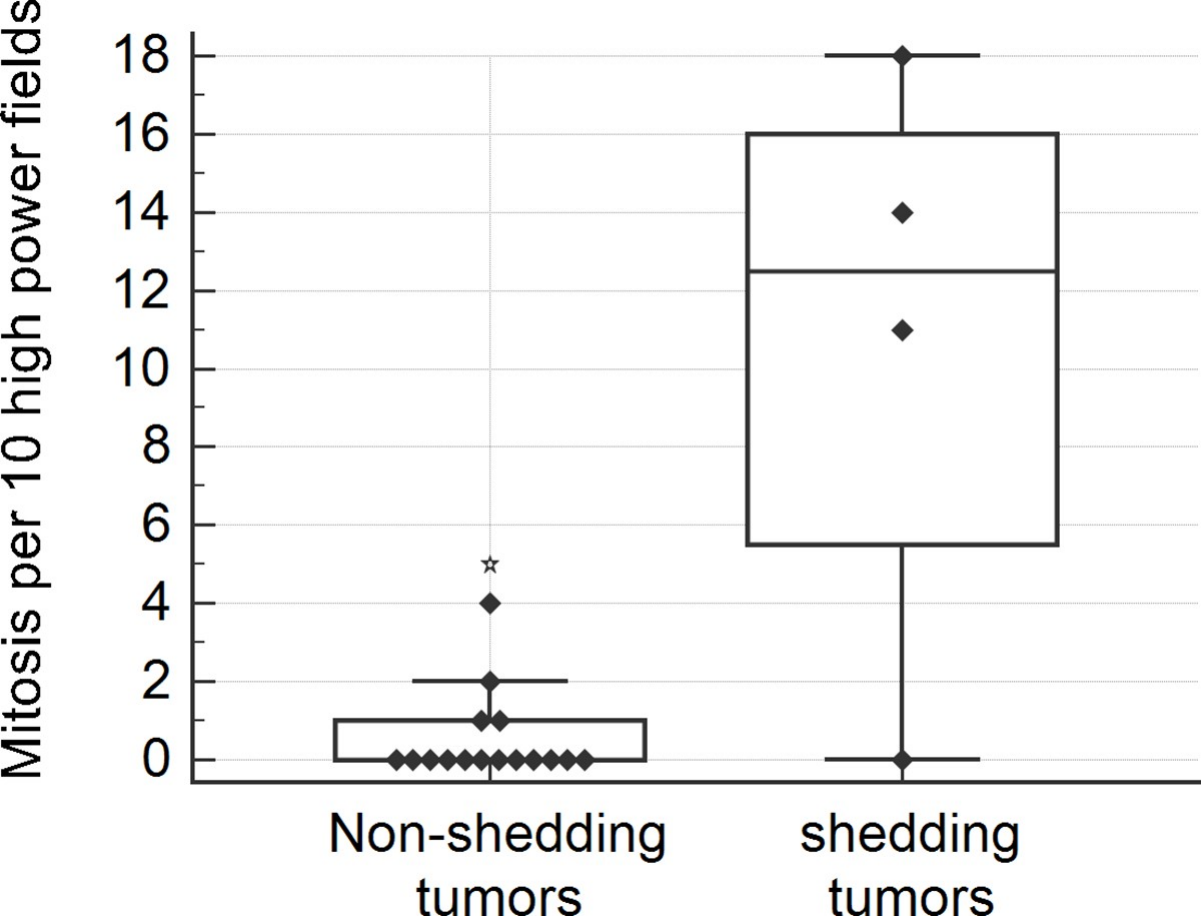
Box and whisker plot of mitotic count per 10 high power fields in non-shedding and shedding tumors.

**Fig 5:**
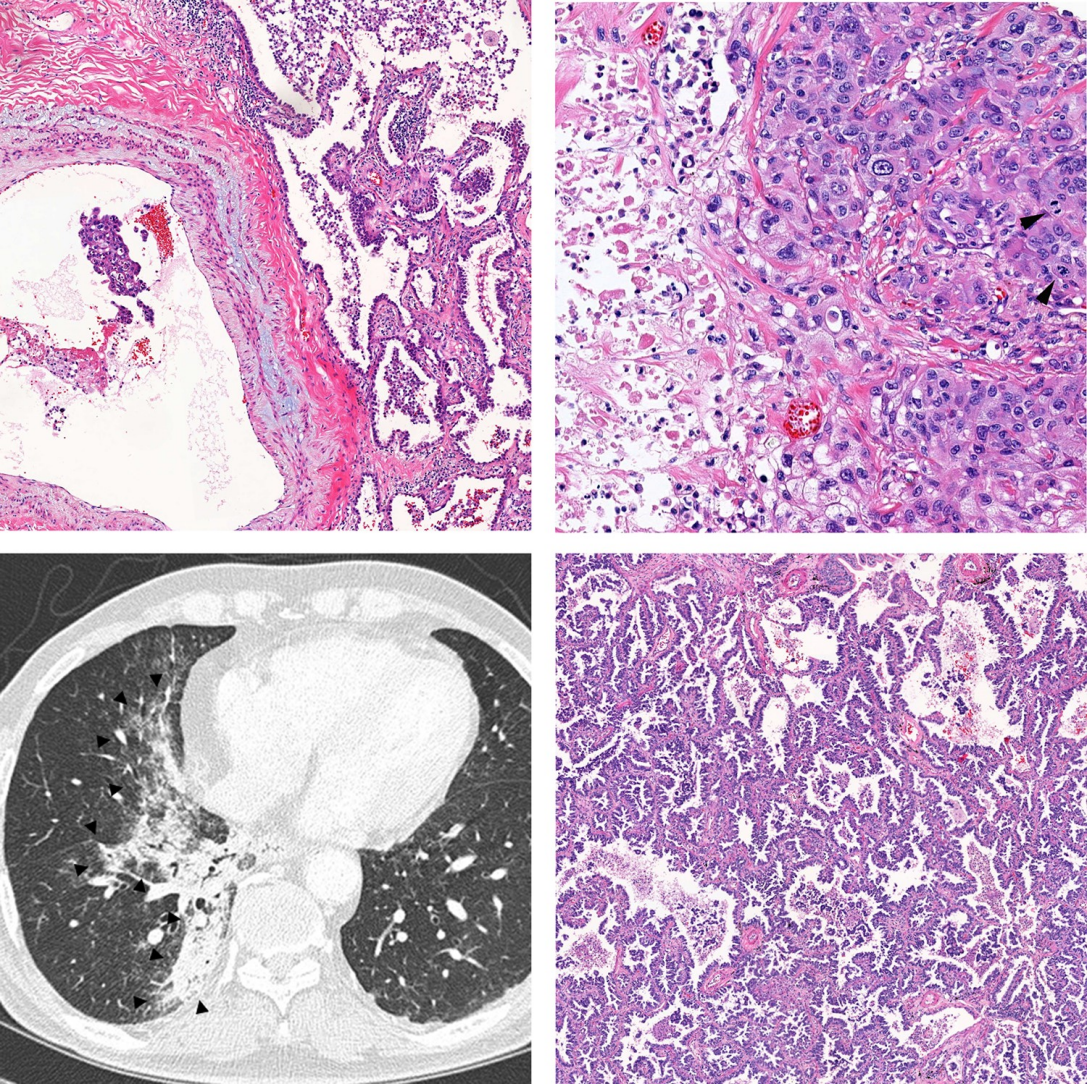
Representative cases with ctDNA shedding in plasma. A-B, A solid predominant adenocarcinoma with EGFR L858R mutation in both tissue and plasma (case no. 35). The tumor showed vascular invasion (A, ×40), necrosis (B; left side of the picture, ×200), cytological atypia, and frequent mitosis (B, arrow heads). C-D, A pneumonic-type adenocarcinoma with KRAS G12V mutation in both tissue and plasma (case no. 13). Large tumor in the right lower lobe on chest CT image is delineated by arrow heads (C). The tumor showed adenocarcinoma with mixed papillary and lepidic pattern (D, ×40).

The presence of ctDNA in plasma was significantly associated with higher pathological tumor stage (*p* = 0.008), nodal metastasis (*p* = 0.013), solid adenocarcinoma pattern (*p* = 0.002), and tumor necrosis (*p* = 0.002) in tissue specimens (Table 5). Higher TNM stage (*p* = 0.053), vascular invasion by tumor (*p* = 0.08) and severe cytological atypia (*p* = 0.053) were marginally associated with ctDNA shedding. In addition, tumors shedding ctDNA in plasma had significantly larger tumor diameter (median [Q1-Q3], 6.8 [5.2–8.6] cm vs. 2.3 [1.90–3.2] cm; *p* = 0.002), tumor volume (median [Q1-Q3], 81.7 [62.3~142.2] cm^3^ vs. 5.5 [2.2–8.7] cm^3^; *p* = 0.002), and higher mitotic rate (median [Q1-Q3], 12.5 [2.8–17] vs 0 [0–1]; *p* = 0.018) than non-shedding tumors (Table 5). In box plots of maximal tumor diameter (Fig 2) and tumor volume (Fig 3), all tumors larger than 4cm in maximal diameter or 25 cm^3^ in volume shed ctDNA in plasma, while tumors smaller than the cutoff did not. In box plot of mitotic rate (Fig 4), tumors with more than 6 mitoses per 10 HPFs were more likely to shed ctDNA. The presence of ctDNA in plasma was not related to other clinicopathological feautres such as sex, pleural invasion, lymphatic invasion, and gross type of the tumor. Among four tumors shedding ctDNA, three cases were solid predominant adenocarcinoma with tumor necrosis, vascular invasion, severe cytological atypia, and lymph node metastasis (Fig 5A and 5B). One case was a 9 cm-sized pneumonic type adenocarcinoma that did not show necrosis, lymphovascular invasion, and node metastasis (Fig 5C and 5D).

**Table 5:** Clinicopathological parameters according to the mutant ctDNA in EGFR or KRAS-mutant lung adenocarcinoma (n = 21).

Clinicopathological features	Mutant ctDNA in plasma				
	Present (n = 4)	Absent (n = 17)	*P*	Odds ratio	95% CI
Categorical variables*					
Sex			1.0	1.125	0.127–9.943
Male	2 (50.0%)	9 (52.9%)			
Female	2 (50.0%)	8 (47.1%)			
Tumor stage			0.028	22.50	1.510–335.338
pT1, T2	1 (25.0%)	15 (88.2%)			
pT3, T4	3 (75.0%)	2 (11.8%)			
Nodal stage†			0.016	42.0	2.010–877.471
pN0	1 (25.0%)	14 (93.3%)			
pN1, N2	3 (75.0%)	1 (6.7%)			
TNM stage			0.053	14.0	1.057–185.492
Stage 1, 2	1 (25.0%)	14 (82.4%)			
Stage 3, 4	3 (75.0%)	3 (17.6%)			
Pleural invasion			1.0	1.083	0.087–13.538
Absent	3 (75.0%)	13 (76.5%)			
Present	1 (25.0%)	4 (23.5%)			
Vascular invasion			0.080	16.0	0.959–267.033
Absent	2 (50.0%)	15 (94.1%)			
Present	2 (50.0%)	1 (5.9%)			
Lymphatic invasion			1.0	1.556	0.117–20.610
Absent	3 (75.0%)	14 (82.4%)			
Present	1 (25.0%)	3 (17.6%)			
Solid predominant pattern			0.003	81.667	2.7249- 2447.567
No	1 (25.0%)	17 (100%)			
Yes	3 (75.0%)	0 (0%)			
Tumor necrosis			0.012	48.0	2.311–997.176
Absent	1 (25.0%)	16 (94.1%)			
Present	3 (75.0%)	1 (5.9%)			
Cytological atypia			0.053	14.0	1.057–185.492
Mild to moderate	1 (25.0%)	14 (82.4%)			
Severe	3 (75.0%)	3 (17.6%)			
Gross type of the tumor			1.0	0.80	0.066–9.669
Solid	3 (75.0%)	12 (70.6%)			
Subsolid	1 (25.0%)	5 (29.4%)			
Continuous variables (median [Q1-Q3]) ‡					
Maximal tumor diameter (cm)	6.8 (5.2–8.6)	2.3 (1.9–3.2)	0.002		
Tumor volume from chest CT (cm^3^)	81.7 (62.3–142.2)	5.5 (2.2–8.7)	0.002		
Mitotic count per 10 HPFs	12.5 (2.8–17)	0 (0–1)	0.018		

* Fisher’s exact test with two-sided *P* values

†Lymph node dissection was performed in 19 patients.

‡ Mann-Whitney *U* test

In subgroup analysis, detection of EGFR mutated ctDNA was significantly associated with nodal metastasis (*p* = 0.029), vascular invasion (*p* = 0.029), solid adenocarcinoma pattern (*p* = 0.010), and tumor necrosis (*p* = 0.010) of primary tumor (Table 6). In addition, tumors shedding EGFR mutant ctDNA in plasma had significantly larger tumor diameter (median [range], 5.45 [4.9–6.0] cm vs. 2.5 [0.8–3.5] cm; *p* = 0.027), tumor volume (median [range], 64.0 [44.60~83.40] cm^3^ vs. 5.45 [0.48–21.80] cm^3^; *p* = 0.027), and higher mitotic rate (median [range] per 10 HPFs, 16 [14–18] vs 0 [0–5]; *p* = 0.009) than non-shedding tumors (Table 6). However, detection of KRAS mutated ctDNA was not significantly associated with any clinicopathological features.

**Table 6:** Subgroup analysis of clinicopathological parameters according to the mutant ctDNA in EGFR-mutant lung adenocarcinoma (n = 15).

Clinicopathological features	Mutant EGFR ctDNA in plasma				
	Present (n = 2)	Absent (n = 13)	*P*	Odds ratio	95% CI
Categorical variables*					
Sex			0.467	1.333	0.894–1.989
Male	0 (0%)	7 (53.8%)			
Female	2 (100%)	6 (46.2%)			
Tumor stage			0.133	0.071	0.011–0.472
pT1, T2	1 (50.0%)	13 (100%)			
pT3, T4	1 (50.0%)	0 (0%)			
Nodal stage†			0.029	3.0	0.606–14.864
pN0	0 (0%)	12 (92.3%)			
pN1, N2	2 (100%)	1 (7.7%)			
TNM stage			0.257	12.0	0.384–374.837
Stage 1, 2	1 (50%)	12 (92.3%)			
Stage 3, 4	1 (50%)	1 (7.7%)			
Pleural invasion			0.371	5.50	0.235–128.968
Absent	1 (50%)	11 (84.6%)			
Present	1 (50%)	2 (15.4%)			
Vascular invasion			0.029	3.0	0.606–14.864
Absent	0 (0%)	12 (92.3%)			
Present	2 (100%)	1 (7.7%)			
Lymphatic invasion			0.371	5.5	0.235–128.968
Absent	1 (50%)	11 (84.6%)			
Present	1 (50%)	2 (15.4%)			
Solid predominant pattern			0.010	Not applicable	
No	0 (0%)	13 (100%)			
Yes	2 (100%)	0 (0%)			
Tumor necrosis			0.010	Not applicable	
Absent	0 (0%)	13 (100%)			
Present	2 (100%)	0 (0%)			
Cytological atypia			0.057	2.0	0.751–5.329
Mild to moderate	0 (0%)	11 (82.6%)			
Severe	2 (100%)	2 (15.4%)			
Gross type of the tumor			0.524	0.80	0.587–1.091
Solid	2 (100%)	8 (61.5%)			
Subsolid	0 (0%)	5 (38.5%)			
Continuous variables (median [range]) †					
Maximal tumor diameter (cm)	5.45 (4.9–6.0)	2.5 (0.8–3.5)	0.027		
Tumor volume from chest CT (cm^3^)	64.0 (44.60–83.40)	5.45 (0.48–21.80)	0.027		
Mitotic count per 10 HPFs	16 (14–18)	0 (0–5)	0.009		

* Fisher’s exact test with two-sided *P* values

† Mann-Whitney *U* test

## Discussion

cfDNA is the extracellular DNA present in the blood that is released from normal as wells as tumor cells, fetuses [12], and viruses. ctDNA represents a small fraction of cfDNA, which is originated from tumor cells. It is considered to be shed when the phagocytic macrophages are exhausted to scavenge ctDNA fragments produced during tumor apoptosis or necrosis [6]. Living tumor cells actively secret ctDNA via exosomes to communicate with distant tissue [7, 13]. Tumor cells circulating in the blood can also contribute to release ctDNA [14]. Tumor DNA contains unique somatic mutation, so mutant DNA in the blood is highly tumor-specific and can be used as tumor biomarkers. Clinical test for detecting mutated tumor DNA in blood sample (liquid biopsy) has become an important goal in NSCLC because obtaining adequate tumor tissues for molecular analysis is not always feasible in advanced lung cancer patients.

The sensitivity of plasma EGFR mutation detection using PCR methods was recently reported to be 10% to 22.2% in stage I-IIIA lung cancer [15, 16]. In this study, the concordance rate between plasma obtained before surgery and resected tissue was 63.8% in EGFR and 88.9% in KRAS; sensitivity was 13.3% in EGFR and 33.3% in KRAS. This concordance and sensitivity is low in our study compared to a similar study by Han *et al*. [17] that used an advanced NSCLC cohort (concordance rate 82.0% for EGFR and 85.9% for KRAS; sensitivity 66.7% for EGFR and 50.0% in KRAS). The low concordance and sensitivity in our study are likely due to false negatives on plasma analysis. Considering that both studies used the same plasma detection flatform (PANAmutyper^TM^ R), tumor biology might determine the sensitivity. Our study cohort included earlier tumor stages, so tumors in our study might be less likely to release ctDNA compared to Han *et al*.’s cohort.

In the present study, sensitivity of ctDNA detection was higher in KRAS than EGFR. However, this is hard to generalize because the number of KRAS mutated lung adenocarcinoma is much smaller (n = 6) than EGFR mutated adenocarcinoma (n = 15) in this study cohort. Further study with larger sample size including more KRAS mutated tumors is needed to clarify this.

Two studies compared ctDNA obtained before surgery and tumor tissue DNA using NGS in surgically resected lung cancer patients [8, 18]. Since NGS can detect various mutations in many genes, we calculated the plasma detection sensitivity of EGFR and KRAS mutations in those studies for comparison with our results. The sensitivity of plasma EGFR mutation detection in Chen’s and Guo’s studies were 56.5% and 63.2%, respectively; the sensitivity of plasma KRAS mutation detection was 60.0% and 50.0%. The specificity of plasma EGFR mutation detection was 72.7% and 75.0%; the specificity for KRAS, 95.0% and 96.0%. The sensitivity for plasma detection of EGFR and KRAS mutations in these two studies using NGS was superior to our study, but the specificity was lower. The authors suggested that false-positive plasma result could be attributed to intratumoral heterogeneity. However, intratumoral heterogeneity is usually related to tumor progression, recurrence, and resistance to therapy [19]. In addition, except for T790M and C797S mutations in EGFR, EGFR and KRAS mutations in NSCLC occur early in carcinogenesis and intratumoral heterogeneity of both genes is rare [20, 21]. Therefore, validation with tissue samples is crucial when performing plasma genotyping, especially in treatment-naïve patients and in the initial phases of NSCLC before progression.

There are factors affecting false negativity in ctDNA analysis. In the pre-analytical phase, contamination with normal DNA from leukocyte lysis during blood clotting dilutes ctDNA and leads to detection failure. Using plasma rather than serum, collecting blood in a K_2_ EDTA tube, and isolating plasma within 6 hours after sampling can minimize pre-analytical error [22]. In the analytical phase, the limit of detection of ctDNA assay is an important determinant of sensitivity. Generally, NGS-based methods are more sensitive than PCR-based methods for detecting low levels of DNA. However, even deep sequencing by NGS could not detect ctDNA in 10% of cases in a recent report [23]. This implies that detection of ctDNA in plasma depends on other factors such as ctDNA shedding. In the post-analytical phase, degree of cfDNA shedding determines the sensitivity of ctDNA tests. For example, ctDNA can easily be detected in tumors shedding ctDNA more heavily into plasma than can be cleared by the liver and kidney.

In advanced NSCLC, ctDNA detection is related to tumor burden as well as extrathoracic lymph node and bone metastasis [24]. In early-stage NSCLC, Abbosh *et al*. showed that non-adenocarcinoma histology, lymphovascular invasion, and high Ki-67 proliferation index were independent predictors of ctDNA detection [9]. Chen *et al*. reported that higher stage (stage II rather than I) and GGO-dominant status led to a significantly higher cfDNA level [8].

In this study, ctDNA shedding in surgically resected adenocarcinoma was related to higher pathological T stage, nodal metastasis, solid adenocarcinoma pattern, tumor necrosis, high mitotic rate, large pathological tumor size, and large tumor volume on CT. In subgroup analysis among EGFR mutated adenocarcinoma, ctDNA was significantly associated with nodal metastasis, vascular invasion, solid adenocarcinoma pattern, and tumor necrosis, high mitotic rate, large pathological tumor size, and large tumor volume on CT. However, KRAS mutated ctDNA was not significantly associated with any clinicopathological features, probably due to small number of KRAS mutated tumor. On case reviews, all three cases of solid predominant adenocarcinoma released ctDNA into plasma and most showed tumor necrosis, vascular invasion, frequent mitosis, severe cytological atypia, and nodal metastasis. However, one case with a large primary tumor size and volume also released ctDNA, despite the absence of other aggressive features. Thus, primary tumor size and volume are an important predictor of ctDNA shedding. Abbosh *et al*. also showed that tumor burden was correlated with mean plasma variant allele frequency [9]. In the present study, all tumors larger than 4 cm in maximal diameter or 25 cm^3^ in volume shed enough ctDNA in plasma to be detected using a PCR-based method with a 0.1% of limit of detection.

This study has some limitations. First, the sample size of this study is quite small, so we could not perform in-depth analysis. Collecting sufficient preoperative blood samples from each NSCLC patient is difficult at a single institute. Further studies with a larger sample size are needed to define pathophysiology of ctDNA shedding. Second, the ctDNA detection method of our study (PNA clamping-assisted PCR) is less sensitive and less informative than NGS-based methods. However, PCR-based plasma testing is cost-effective, fast, and simple to interpret in daily practice, and thus we believe these findings have more practical value in the real world setting.

In summary, this study evaluated EGFR and KRAS mutations in matched tissue and preoperative plasma samples from surgically resected NSCLC and assessed the clinicopathological predictors for ctDNA release in plasma. Shedding of ctDNA from tumor tissue into plasma is low (overall sensitivity 19.0%) in surgically resected NSCLC patients. Our results suggested that primary tumor size and volume as well as aggressive histologic features such as solid adenocarcinoma pattern, necrosis, and frequent mitosis could predict ctDNA shedding in pulmonary adenocarcinoma.

## Supporting information

S1 Dataset (XLSX)
